# Relaxin combined with transarterial chemoembolization achieved synergistic effects and inhibited liver cancer metastasis in a rabbit VX2 model

**DOI:** 10.1007/s00432-024-05864-6

**Published:** 2024-07-02

**Authors:** Fuquan Wang, Licheng Zhu, Fu Xiong, Bin Chai, Jihua Wang, Guofeng Zhou, Yanyan Cao, Chuansheng Zheng

**Affiliations:** 1 Department of Radiology, Union Hospital, Tongji Medical College, Huazhong University of Science and Technology, Wuhan, 430022 Hubei China; 2 Hubei Province Key Laboratory of Molecular Imaging, Wuhan, 430022 Hubei China

**Keywords:** Relaxin, Transarterial chemoembolization, Doxorubicin, Liver cancer, Metastasis, HIF-1α

## Abstract

**Objective:**

To explore the effect and mechanism of relaxin (RLX) in the growth and metastasis of livercancer after combination treatment with transarterial chemoembolization (TACE).

**Materials and methods:**

HCCLM3 and Huh-7 cells were adopted to evaluate the effect of tumor proliferation, migration, and invasion after RLX administration in vitro. The rabbit VX2 model was used to evaluate the biosafety, doxorubicin penetration, local tumor response, tumor metastasis, and survival benefit of RLX combined with TACE treatment.

**Results:**

RLX did not affect the proliferation, migration, or invasion of HCCLM3 and Huh-7 cells, and the expression of E-cadherin and HIF-1α also remained unchanged while the MMP-9 protein was upregulated in vitro. In the rabbit VX2 model, compared to the normal saline group (NS), RLX group (RLX) and TACE mono-therapy group (TACE), the group that received TACE combined with RLX (TACE + RLX) showed an improved local tumor response and survival benefit. Furthermore, TACE combined with RLX was found to reduce tumor metastasis. This combination therapy reduced the fibrotic extracellular matrix in the tumor microenvironment, allowing for better penetration of doxorubicin, improved infiltration of CD8+ T cells and affected the secretion of cytokines. Additionally, RLX combined with TACE was able to decrease the expression of HIF-1α and PD-L1. The biosafety of TACE combined with RLX was also confirmed.

**Conclusion:**

RLX synergized with TACE by mitigating the fibrotic extracellular matrix and tumor hypoxic microenvironment, improving the therapeutic effect and inhibiting metastasis during the treatment of liver cancer.

**Supplementary Information:**

The online version contains supplementary material available at 10.1007/s00432-024-05864-6.

## Introduction

Liver cancer ranked sixth in global incidence and as the fourth leading cause of cancer mortality worldwide in 2022 (Ganesan and Kulik 2023). Hepatocellular carcinoma (HCC) is the most common type of primary liver cancer. In cases of early-stage HCC, the guidelines recommend primary treatment options including liver resection, transplantation, or ablation (Marron et al. 2022; Jiang et al. 2019). For advanced-stage HCC, transarterial chemoembolization (TACE) is recommended because the results from several large randomized controlled trials confirmed a survival benefit with TACE compared to patients receiving supportive care (Llovet et al. 2002; Yang and Heimbach 2020).

Even though TACE has been used extensively in the therapy of liver cancer, it also faces the challenges of tumor growth and metastasis in patients after treatment (West and Jin 2015). The effect of TACE is strongly related to the highly desmoplastic tumor microenvironment (TME), which is mainly manifested in the extracellular matrix (ECM) with a large amount of fibrotic stroma (Tan et al. 2023, Barbier et al. 2021; Doemel et al. 2022). The formation of such ECM is due to the characteristics of the liver cancer itself, and secondary hypoxia due to the blockade of the arteries supplying the liver cancer by the TACE treatment, which enhances the expression of HIF-1α and causes fibrotic changes in the ECM (Roy et al. 2023; Desert et al. 2023; Daniele et al. 2014). These alterations not only prevent the penetration of chemotherapy drugs such as doxorubicin (DOX) but also limit the infiltration of CD8+ T cells into the tumor tissue (Ying et al. 2023). Therefore, reducing the fibrotic ECM within HCC has the potential to enhance the antitumor outcome of chemotherapy administered during a TACE procedure.

Relaxin (RLX) is a hormone with antifibrotic properties that inhibits profibrotic cytokine-mediated abnormal fibroblast proliferation, differentiation and matrix production by binding to its primary receptor RXFP1 (relaxin family peptide receptor 1) (Zhou et al. 2021b; Samuel et al. 2022). Previous research have demonstrated that RLX has the ability to upregulate matrix metalloproteinase (MMP), including MMP-2 and MMP-9, and decrease the formation of fibrotic ECM in the treatment of pancreatic cancer and prostate cancer, resulting in positive therapeutic outcomes (Mardhian et al. 2018; Feng et al. 2010). However, research conducted on breast cancer models has reported contrasting findings, suggesting that RLX may actually promote breast tumor growth and metastasis (Binder et al. 2014, 2002). Considering the differences in the treatment of different types of tumors, the exploration the role of RLX in liver cancer is of significance.

This study investigated the cytotoxicity of RLX and its function in hypoxia using liver cancer cell lines. Additionally, we examined the safety and effectiveness of combining TACE with RLX in the rabbit VX2 tumor model. Our findings further confirmed that the combination of TACE and RLX can exhibit a synergistic treatment effect and inhibit the metastasis of liver cancer.

## Materials and methods

### Cell culture, reagents and animals

HCCLM3, Huh-7, and LO2 cells of human origin were cultivated using Dulbecco’s Modified Eagle’s Medium (Servicebio, Wuhan, China) with high levels of glucose, along with the addition of 10% fetal bovine serum, 100 mg/ml penicillin, and 100 mg/ml streptomycin. The cultured cells were then subjected to incubation at a temperature of 37 ℃ under two different atmospheric conditions: normoxia, consisting of a humidified atmosphere with 21% O2, and hypoxia, with a humidified atmosphere of 5% O2. RLX was acquired from Prospec Inc. (Hamadan, Israel). The antibodies used in the experiments included a monoclonal antibody against human GAPDH (Antgene, Wuhan, China), and antibodies against actin (Antgene, Wuhan, China), E-cadherin (Proteintech Group Inc., Chicago, USA), HIF-1α (GeneTex, Southern California, USA), and MMP-9 (sigma, Milan, Italy).

Adult male New Zealand white rabbits weighing 2.0–2.5 kg were purchased from the Laboratory Animal Center of Huazhong University of Science and Technology. The institute’s Ethics Committee granted authorization for all procedures, which complied with the National Institutes of Health Guide for the Care and Use of Laboratory Animals.

### Cytotoxicity assays

The CCK-8 method was used to evaluate the cytotoxicity of RLX following the manufacturer’s instructions. Briefly, the HCCLM3, Huh-7, and LO2 cells were cultured in 96-well plates and supplemented with CCK-8 solution (10 μl/well) and RLX at concentrations of 0, 0.01, 0.1, and 1 μM. Cells were incubated under the described conditions for 1–4 h to confirm the cytotoxic data. Each experiment was repeated three times, and absorbances were detected at 450 nm with a microplate reader.

### Wound healing and Transwell assays

The impact of RLX on HCCLM3 and Huh-7 cell migration was ascertained by seeding the cells into six-well plates and incubating them in a serum-free RPMI-1640 medium. After 1 day, when the cells had grown to 80–90% confluence, we gently scratched the monolayer with a 200-μl pipette tip perpendicular to the bottom of the plate. Next, the cell debris was removed by washing three times with PBS. The remaining cells were then cultured in medium without FBS. After scratching, images were captured at 0 and 24 h under a light microscope at × 100 magnification. Wound closure was examined by the ImageJ program (version 1.51; National Institutes of Health).

Transwell cell invasion assays were conducted with Transwell chambers (12-μm pore size, Corning). Matrigel was added to the apical side of the Transwell membrane to form a uniform thin layer of gel. The cells were seeded at a concentration of 1.0 × 105 cells per chamber in the upper chamber. Serum-free medium was used for seeding the cells in the upper chamber, while the lower chamber was filled with complete culture medium containing 20% FBS. The cells were then incubated for a period of 24 h to assess their invasion capabilities.

### Western blot analysis

The HCCLM3 and Huh-7 cells were separated into four groups and administered PBS (Control group) or RLX at concentrations of 0.01, 0.1, or1 μM in normoxic or hypoxic conditions. Following 24 h of incubation, we obtained total proteins from the HCCLM3 and Huh-7 cells utilizing a comprehensive extraction kit. The proteins obtained were analyzed through gel electrophoresis, transferred onto PVDF membranes and underwent a 1-h incubation with 5% BSA. Subsequently, primary antibodies against MMP-9, E-cadherin, and actin were applied and incubated overnight at 4 ℃. Afterward, a secondary antibody was utilized and incubated for 1 h at room temperature. Ultimately, visualization of the protein bands was accomplished using a chemiluminescence system (PerkinElmer, Waltham, MA, USA).

### Immunofluorescence analysis

The HCCLM3 and Huh-7 cells were separated into four groups and administered PBS (Control group) or RLX at concentrations of 0.01, 0.1, or 1 μM in normoxic or hypoxic conditions. A 4% paraformaldehyde solution was used to fix the cells, and then they were washed with PBS three times. To prevent nonspecific binding of antibodies, 10% goat serum was added as a blocking agent. Following that, the cells were subjected to staining using the aforementioned HIF-1α antibodies. For visualization, Alexa Fluor 488-conjugated donkey anti-rabbit IgG secondary antibody (Antgene, Wuhan, China) was employed. To stain the nuclei, 4,6-diamidino-2-phenylindole (DAPI) was used. Finally, the cells were observed under a fluorescence microscope (Olympus, Japan) after sealing with antifade mounting medium.

### Animal tumor model

The establishment process of the rabbit VX2 liver cancer model was conducted in the following manner: First, solid tumors were obtained from tumor‐bearing rabbits that were injected with VX2 tumor cell (1 × 10^6^) suspension in the right thigh muscle. The tumors were then carefully dissected into small tissue cubes, each measuring 1 mm^3^ in size. Second, the rabbits were then anesthetized with an intravenous injection of 2% pentobarbital sodium solution at 0.3 ml/kg, and tumor cubes were implanted into the left lobe of the liver under sterile conditions through a partial abdominal incision. Third, all rabbits were injected intramuscularly with penicillin after implantation for 3 days to prevent postoperative infection. After two weeks, when the tumor reached a diameter of 15–20 mm on computed tomography (CT) scanning, the rabbits were randomly assigned to the corresponding treatment groups for the experiment.

### Groups and intervention procedure

Forty rabbits with liver VX2 tumors were divided into four groups at random: group 1 received normal saline (3 ml) through tumor feeding artery infusion as a control (NS group); group 2 received RLX (5 mg/ml, about 14 μg in total) through tumor feeding artery (RLX group); group 3 received doxorubicin (DOX, 4 mg/kg)/lipiodol emulsion (3 ml) and gelfoam embolization through tumor feeding artery (TACE group); group 4 received RLX (5 mg/ml, about 14 μg in total) combined with doxorubicin/lipiodol emulsion (3 ml) and gelfoam embolization through the tumor feeding artery (RLX + TACE group). The transcatheter procedures were conducted as follows: first, a 4-F vascular sheath was implanted into the rabbit right femoral artery through an open puncturation; second, a 4-F visceral catheter (Cordis, Cardinal Health, USA) was introduced into the celiac axis under the guidance of digital subtraction angiography; third, we introduced a 2.7-F microcatheter (Terumo, Japan) into the left hepatic artery. Subsequent procedures were conducted according to the different groups mentioned above. Auxiliary embolization was then performed with gelfoam particles (350–500 μm) until anterograde flow in the feeding vessel was completely arrested (Fig. S3).

To assess the rabbits’ tumor development, dynamic contrast-enhanced CT was performed 7 days post treatment. The tumor’s diameter was measured, and subsequently, its volume was computed using the formula V = a ∗ b2/2, where ‘a’ and ‘b’ denote the maximum and minimum diameters, respectively. Determining the tumor growth rate required a comparison of the volume before and after at the 7 days post-treatment.

### Hematoxylin‐eosin staining and immunohistochemical staining

Histological analysis was conducted on tumor tissues, which had been immersed in a 10% formalin solution, embedded in paraffin, and subsequently sectioned. These sections underwent staining methods including hematoxylin–eosin (H&E), TUNEL, Ki-67, and Masson for detailed histological analysis. Immunohistochemical or immunofluorescence staining was performed using monoclonal or polyclonal antibodies against CD8+ T (Dako, Copenhagen, Denmark), Foxp3 + Treg (Servicebio, Wuhan, China), F4/80 (OriGene Technologies, Rockville, USA), CD206 (OriGene Technologies, Rockville, USA), MMP-9 (Servicebio, Wuhan, China), mouse anti-HIF-1α antibody (GeneTex, Southern California, USA), mouse anti-MMP-2 antibody, rabbit anti PD-L1 antibody, rabbit anti α-SMA antibody, and rabbit anti-E-cadherin antibody (Proteintech Group Inc., Chicago, USA). The expression of each index was observed under an Olympus BX50 fluorescence microscope. ImageJ (Media Cybernetics, Rockville, USA) was utilized to evaluate different parameters by analyzing the density and staining intensity of the tissue image regions.

### ELISA

The rabbit’s tissue tumors were collected and disperse the tumor tissue in PBS solution. After centrifugation at 1000*g* for 5 min, collect the supernatant. ELISA kits (BD Biosciences) were used to detect the levels of IL-6, IL-10, and TNF-α in the supernatants.

### Statistical analysis

The researchers conducted statistical analyses utilizing SPSS software (SPSS, version 24.0, Chicago, IL, USA). All data are presented as the mean ± SD and analyzed by independent samples t tests or one-way ANOVA. *p* < 0.05 was considered statistically significant. GraphPad Prism V8.3 (San Diego, CA, USA) was employed for the creation of all illustrations.

## Results

### RLX was not hepatotoxic and did not affect the proliferation, migration, or invasion of HCCLM3 cells and Huh-7 cells cells

**Fig. 1:**
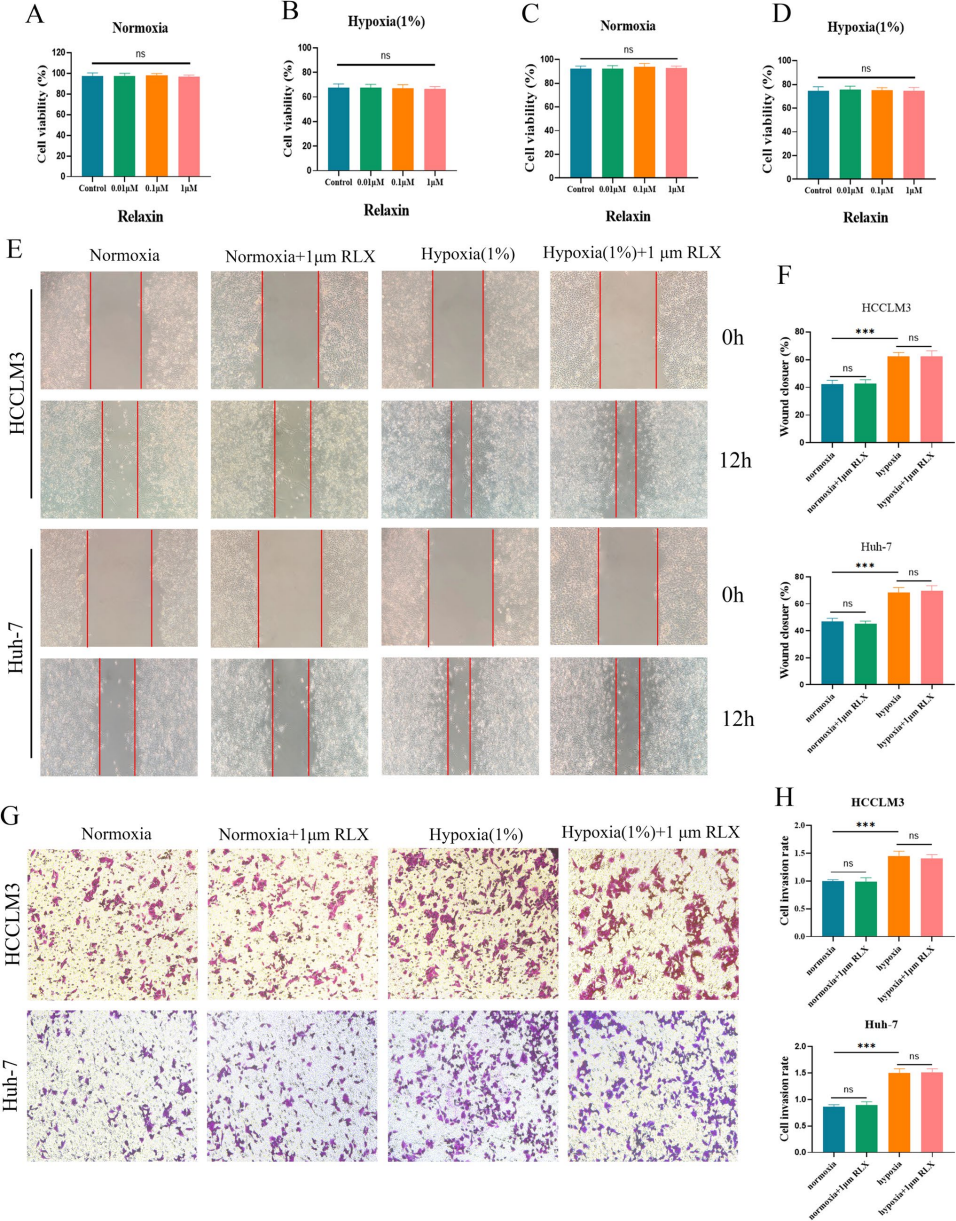
RLX did not affect the proliferation, migration, or invasion of HCCLM3 cells and Huh-7 cells without hepatotoxicity. The hepatotoxicity of RLX (0, 0.01, 0.1, and 1 μM) on normoxia (**A**)/hypoxic (**B**) via CCK-8; the cytotoxicity of RLX (0, 0.01, 0.1, and 1 μM) on normoxia (**C**)/hypoxia (**D**) via CCK-8 on LM3 cells; **E** representative wound healing test images of HCCLM3 cells and Huh-7 cells in each group; **F** quantitative analysis of HCCLM3 cells and Huh-7 cells in the wound healing test; **G** representative transwell assays images of HCCLM3 cells and Huh-7 cells in each group; **H** quantitative analysis of HCCLM3 cells and Huh-7 cells in the transwell assays (means ± SDs; ns > 0.05, *p < 0.05, **p < 0.01, ***p < 0.001). *RLX* relaxin

To investigate the hepatotoxicity of RLX, CCK-8 assays were used to detect the viability of LO2 cells treated with 0.01 μM, 0.1 μM, or 1 μM of RLX. Different concentrations of RLX did not affect the viability of LO2 cells either under normoxia or hypoxia (*p* > 0.05) **(**Fig. 1A, B**)**. Different concentrations of RLX were then used to treat HCCLM3 cells, and the experimental findings indicated that RLX did not have a noteworthy impact on the proliferation of HCCLM3 **(**Fig. 1C, D**)** and Huh-7 cells **(**Fig. S1**)** in both normoxic and hypoxia environments (*p* > 0.05). The results of the wound healing **(**Fig. 1E, F**)** and Transwell-based assays **(**Fig. 1G, H**)** showed that the migratory and invasive properties of HCCLM3 and Huh-7 cells did not change significantly after RLX treatment with different concentrations (*p* > 0.05). However, the migration and invasion of HCCLM3 and Huh-7 cells seemed enhanced in the hypoxia condition compared with normoxia (*p* < 0.001).

### RLX promoted the expression of MMP-9 but without effect on E-cadherin and HIF-1α

**Fig. 2:**
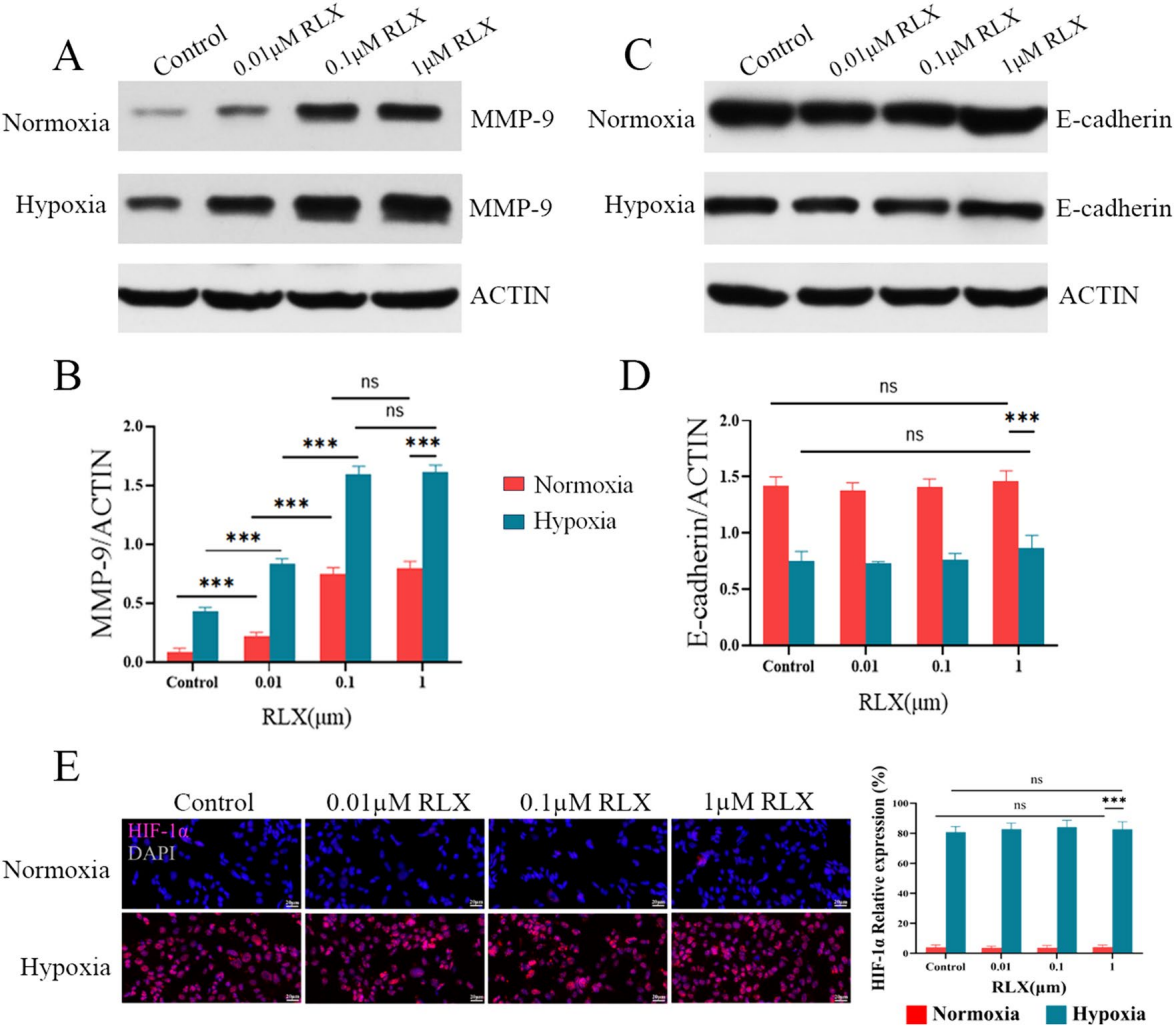
RLX upregulated the expression of MMP-9 but have no impact on the expression of E-cadherin and HIF-1α in HCCLM3 cells. **A** Effects of normoxia/hypoxia and administration RLX (0, 0.01, 0.1, and 1 μM) on MMP-9 expression levels in HCCLM3 cells; **B** quantitative analysis of MMP-9 levels in HCCLM3 cells; **C** effects of normoxia/hypoxia and administration RLX (0, 0.01, 0.1, and 1 μM) on E-cadherin expression levels in HCCLM3 cells; **D** quantitative analysis of E-cadherin levels in HCCLM3 cells; **E** immunofluorescence staining of HIF-1α in each group after RLX administration (0, 0.01, 0.1, and 1 μM) (means ± SDs; ns > 0.05, *p < 0.05, **p < 0.01, ***p < 0.001). *MMP-9* matrix metalloproteinases-9, *HIF-1α* hypoxia induicible factor-1alpha, *RLX* relaxin

This investigation involved Western blot analysis of HCCLM3 **(**Fig. 2A–D**)** and Huh-7 cells (Fig. S2A–D) that underwent treatment with varying doses of RLX. The findings indicated a substantial elevation in the levels of MMP-9 and E-cadherin expression under hypoxia conditions relative to normoxia conditions. Meanwhile, RLX was found to significantly increase the expression of MMP-9 both under normoxia and hypoxia. The degree of promotion at a concentration of 0.1 μM RLX was higher than that at 0.01 μM, but there was no significant difference compared to 1 μM RLX in HCCLM3 cells. It is worth mentioning that different concentrations of RLX did not affect the expression of E-cadherin under either normoxic or hypoxic conditions for both HCCLM3 and Huh-7 cells. In addition, similar results were obtained for the expression of HIF-1α protein in the immunofluorescence assay, which displayed that HIF-1α protein was increased under hypoxic conditions (*p* < 0.001) while remaining unchanged after treatment with different concentrations of RLX (*p* > 0.05) **(**Fig. 2E**)**.

### Inhibit tumor growth, metastases and prolong survival in TACE + RLX group

**Fig. 3:**
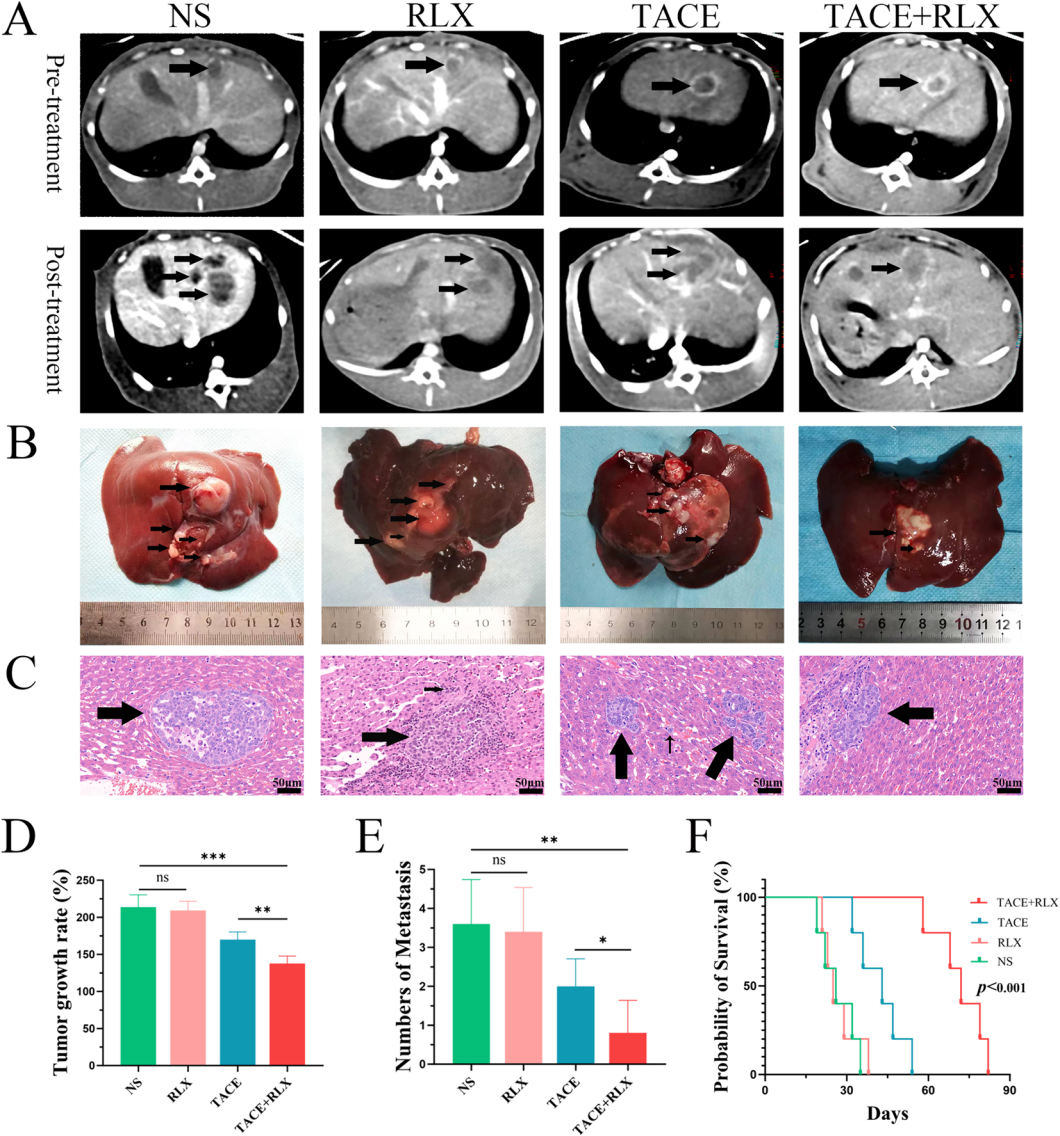
TACE combined with RLX inhibited tumor growth, metastases and prolonged survival in VX2 tumor rabbits. **A** CT enhancement (arterial phase) visualize the tumors in each group before and 7 days after treatment; **B** gross images and metastases (arrow) of liver in each group; **C** H&E staining of liver metastases (arrow); **D** quantitative analysis of tumor growth rate in each group; **E** quantitative analysis of metastasis in each group; **F** Kaplan–Meier survival curves with log-rank tests for rabbits in different groups (means ± SDs; ns > 0.05, *p < 0.05, **p < 0.01, ***p < 0.001). *RLX* relaxin, *TACE* transarterial chemoembolization, *H&E* hematoxylin–eosin, *NS* normal saline

CT was used to evaluate the tumor growth rate in three VX2 model rabbit groups. As illustrated in Fig. 3A, the mean tumor growth rate in the NS group, RLX group, TACE group, and TACE + RLX group was 213 ± 16%, 209 ± 10%, 170 ± 10%, and 137 ± 10%, respectively (Fig. 3D). The TACE + RLX group showed a decreased rate of tumor growth in comparison to the TACE group (*p* < 0.01), RLX group and NS group (*p* < 0.001). The rate of tumor growth between RLX group and NS group have no difference (*p* > 0.05). There were 3.6 ± 1.1 (2–5), 3.4 ± 1.0 (2–5), 2.0 ± 0.7 (1–3), and 0.8 ± 0.8 (0–2) intrahepatic metastases in the NS group, RLX group, TACE group and TACE + RLX group, respectively (Fig. 3B, C, E). Metastatic tumors in the TACE + RLX group were reduced compared to the TACE group (*p* < 0.05) RLX group (*p* < 0.01), and NS group (*p* < 0.01). As shown in Fig. 3F, the treatment of group in the TACE + RLX can significantly improve the survival of rabbits, with a much longer survival time than the other three groups (*p* < 0.001).

### Necrosis rate, and immunohistochemical staining analysis of Ki-67 and TUNEL

**Fig. 4:**
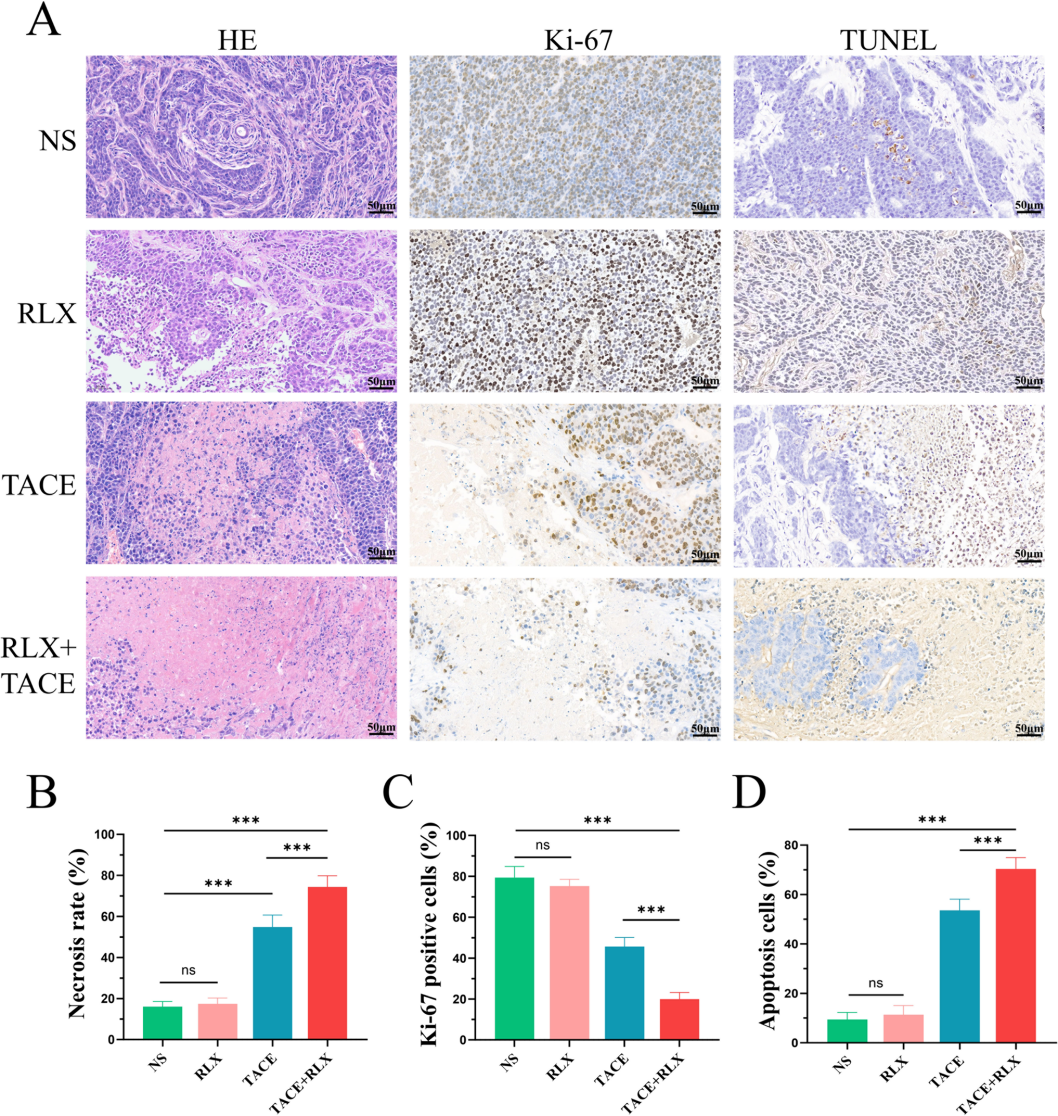
TACE combined with RLX promoted tumor necrosis and apoptosis and inhibited tumor proliferation. **A** H&E staining and immunohistochemistry staining of Ki-67 and TUNEL of liver VX2 tumor tissues after treatments; **B**–**D** quantitative analysis of necrosis rate, Ki‐67 positive cells, and apoptosis cells after treatments in each group (means ± SDs; *p < 0.05, **p < 0.01, ***p < 0.001). *RLX* relaxin, *TACE* transarterial chemoembolization, *H&E* hematoxylin–eosin, *NS* normal saline

The tumor necrosis rate of each rabbit group was evaluated by HE. As displayed in Fig. 4A, B, There were 16 ± 3%, 17 ± 3%, 55 ± 6%, and 74 ± 7% mean necrosis rates in the NS, RLX, TACE, and TACE + RLX groups, respectively. The tumor necrosis rate in the TACE + RLX group was lower than the other three groups (*p* < 0.001). In terms of the percentage of Ki‐67 positive cells, the TACE + RLX group was lower than in the other three groups (*p* < 0.001) (Fig. 4A, C). In contrast, the apoptosis level of tumor cells in the TACE + RLX group was significantly higher than in the TACE group (*p* < 0.001) and NS group (*p* < 0.001) (Fig. 4A, D). It was noteworthy that there were no statistically significant differences in Ki‐67 positive cells and apoptosis level of tumor cells between the NS group and the RLX group (*p* > 0.05).

### Upregulated MMPs protein, degraded collagen tumor tissue and improved DOX penetration in the TACE + RLX group

**Fig. 5:**
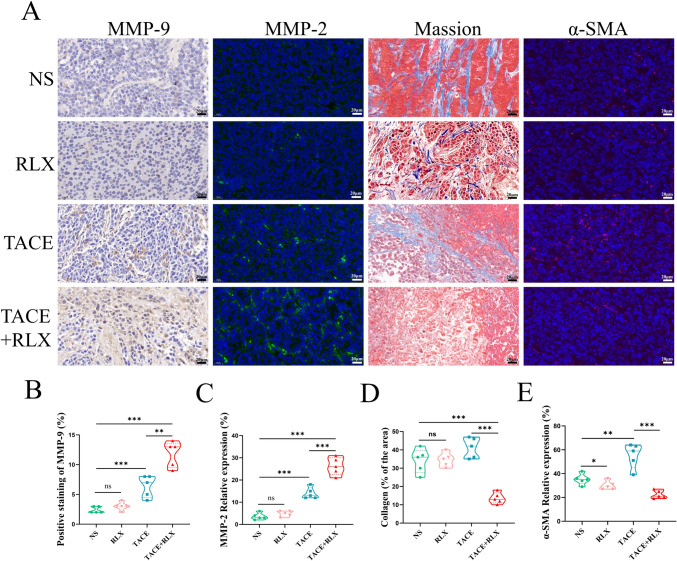
TACE combined with RLX upregulated MMPs and degraded collagen tumor tissue and tumor-associated fibroblasts. **A** Immunohistochemical staining of MMP-9 and immunofluorescence staining of MMP-2 and α-SMA, and Masson staining of collagen-forming fibers; **B** and **C** quantitative analysis of MMP-9 and MMP-2 expression after treatment; **D** quantitative analysis of collagen expression in each group; **E** quantitative analysis of α-SMA expression in each group (means ± SDs; ns > 0.05, *p < 0.05, **p < 0.01, ***p < 0.001). *MMP* matrix metalloproteinases, *RLX* relaxin, *TACE* transarterial chemoembolization, *NS* normal saline

As illustrated in Fig. 5A–C, after RLX treatment alone, the expression of MMP-9 and MMP-2 remained unchanged (*p* > 0.05). After receiving TACE treatment, the expression of MMP-9 and MMP-2 significantly increased, (*p* < 0.001). The combination therapy of TACE and RLX can significantly increase the expression of MMP-9 and MMP-2 in tumor tissue (*p* < 0.001). Considering the collagen degradation capacity of RLX after increasing the expression level of MMPs according to previous research(Zhou et al. 2021a; Lee et al. 2012), Masson staining was therefore conducted. The collagen component was obviously reduced in the combination group compared to the TACE mono-therapy group (*p* < 0.001) RLX group (*p* < 0.001) and control group (*p* < 0.001), either in the necrosis area or in the vicinity of tissue (Fig. 5A, D). In contrast, the collagen component in the TACE group remained without apparent reduction or breaks. What’s more, the collagen in the TACE group showed curl appearance, which was probably due to the induction of necrosis after the TACE procedure. Notably, immunofluorescence staining results showed that the combination therapy of TACE and RLX can inhibit the expression of Tumor-associated fibroblasts (TAF) activation related proteins α-SMA compared with TACE alone (*p* < 0.001) (Fig. 5A, E).

Considering the elevation of MMPs protein expression and degradation of collagen after treatment with RLX, the penetration of DOX was then observed. DOX has natural red fluorescence, so the tumor microvessels were stained with green fluorescence. More infiltration of DOX was observed in the TACE + RLX group than in the TACE group (Fig. S4).

### RLX inhibited HIF-1α expression and slightly increased E-cadherin expression

**Fig. 6:**
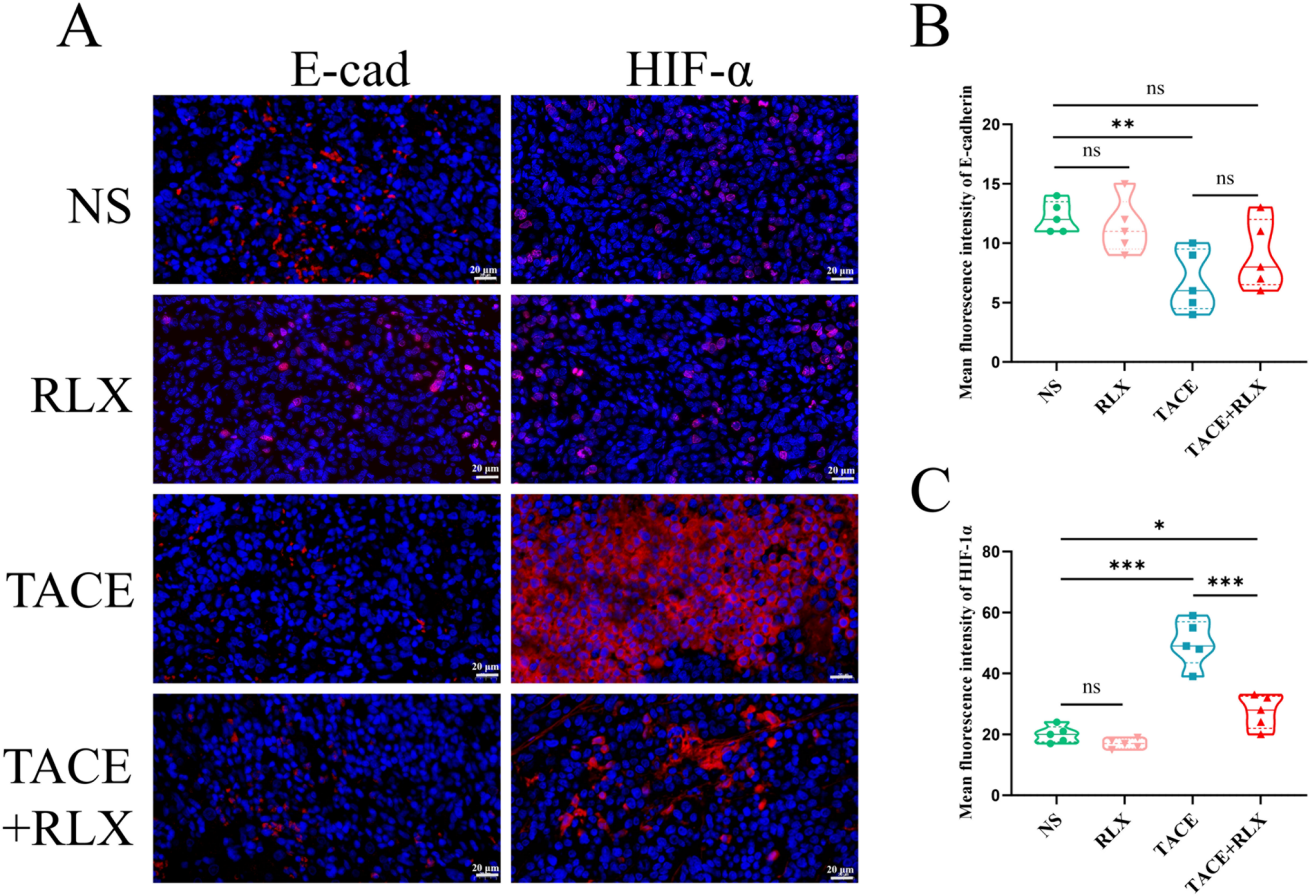
Relaxin inhibited HIF-1α expression and slightly increased E-cadherin expression. **A** Immunofluorescence staining of E-cadherin and HIF-1α in liver cancer tissues in each group; **B** quantitative analysis of protein E-cadherin expression in each group; **C** quantitative analysis of protein HIF-1α expression in each group (means ± SDs; ns > 0.05, *p < 0.05, **p < 0.01, ***p < 0.001). *HIF-1α* hypoxia-inducible factor-1alpha, *RLX* relaxin, *TACE* transarterial chemoembolization, *NS* normal saline

To further clarify if there were alterations of E-cadherin protein expression in tumor tissue, the liver cancer specimens were subjected to immunofluorescence staining (Fig. 6A). Unexpectedly, the results suggested that the expression of E-cadherin protein in the TACE + RLX group did not change significantly (*p* > 0.05) but it increased to a certain degree compared with the TACE group (Fig. 6B). Moreover, there was no significant difference when compared to the RLX group with the NS group (*p* > 0.05), which was higher than the TACE group (*p* < 0.01). In addition, HIF-1α expression was found to be increased after TACE treatment, consistent with our previous findings (Liang et al. 2010). However, when TACE was combined with RLX, the expression of HIF-1α was significantly decreased compared to the group that received TACE alone (*p* < 0.001) (Fig. 6C). This could be attributed to RLX improving the tissue hypoxia caused by TACE embolization. However, there was no significant difference in the expression of HIF-1α between the NS group and the RLX group (*p* > 0.05).

### Improved tumor immune microenvironment after TACE + RLX

**Fig. 7:**
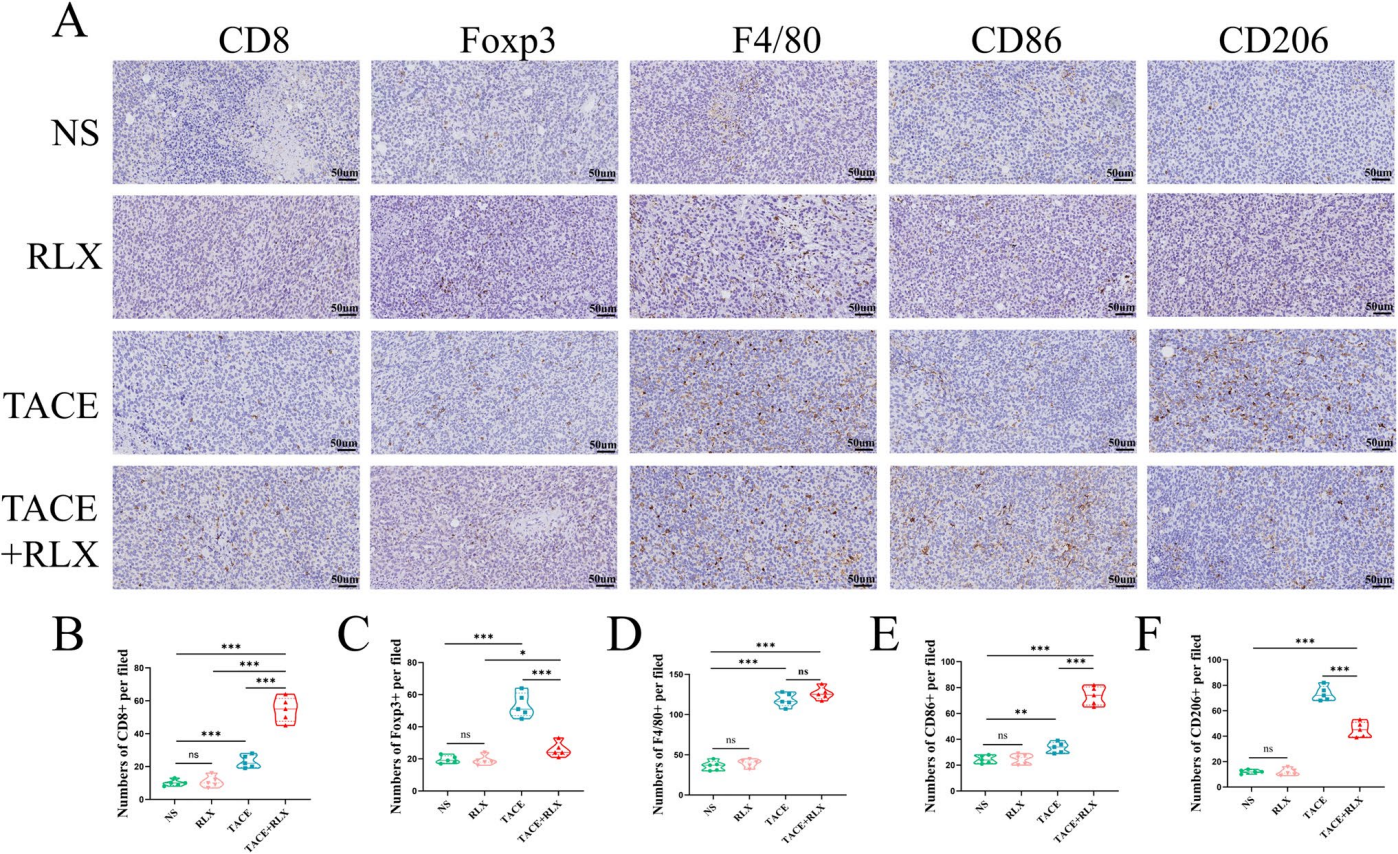
TACE combined with RLX improved the tumor immune microenvironment. **A** Immunohistochemical staining of CD8+ T, Foxp3 + Treg, F4/80 + Macrophage, CD86 + Macrophage, CD206 + Macrophage cells; **B**–**F** quantitative analysis of positive staining rates of above cells (means ± SDs; ns > 0.05, *p < 0.05, **p < 0.01, ***p < 0.001). *RLX* relaxin, *TACE* transarterial chemoembolization, *NS* normal saline

Our research involved the utilization of immunohistochemical staining to examine several key immune cells (due to the unavailability of rabbit antibodies for flow cytometry) (Fig. 7A). Cellular immunity mediated by CD8+ T cells is the primary mechanism of anti-tumor immunity. The results illustrated that the density of CD8+ T cells was significantly higher in the TACE + RLX group than the TACE group (*p* < 0.001), RLX group (*p* < 0.001) and NS groups (*p* < 0.001) (Fig. 7B). The level of Foxp3 + Treg cells infiltration was significantly higher in the TACE group compared to the NS and RLX groups (*p* < 0.001). However, when TACE was combined with RLX, there was a significant reduction in the infiltration level of Foxp3 + Treg cells (*p* < 0.001) (Fig. 7C). RLX did not alter the densities of F4/80 + total macrophages after TACE treatment, which were higher than in the NS and RLX group (*p* < 0.001) (Fig. 7D). Nevertheless, the densities of M1 macrophages (CD86 +) in the TACE + RLX group exhibited a substantial increase in comparison to the other three groups (*p* < 0.001) (Fig. 7E). Conversely, the densities of M2 macrophages (CD206 +) observed a marked decline in the TACE + RLX group compared to the TACE group and RLX group (*p* < 0.001) (Fig. 7F). Remarkably, the tissue of the control group rarely showed infiltrated immune cells, demonstrating an immunologically excluded tumor type.

**Fig. 8:**
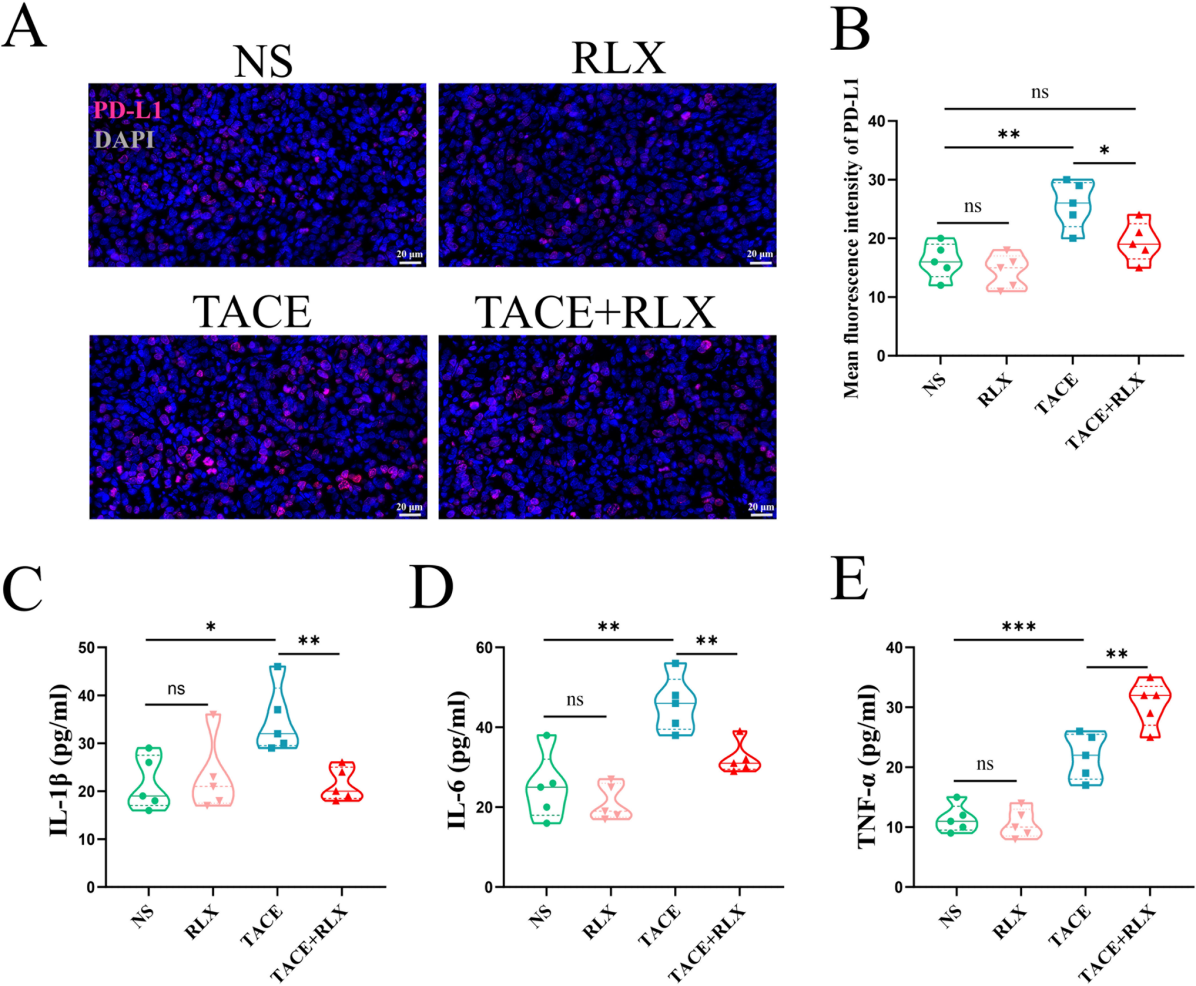
The level of PD-L1 expression and cytokine secretion in each group. **A** immunofluorescence staining of PD-L1 in each group; **B** quantitative analysis of PD-L1 expression after treatment; **C**, **D**, and **E** quantitative analysis of the secretion levels of IL-1β, IL-6 and TNF-α in each group by ELISA (means ± SDs; ns > 0.05, *p < 0.05, **p < 0.01, ***p < 0.001). *RLX* relaxin, *TACE* transarterial chemoembolization, *NS* normal saline

The levels of PD-L1 expression in tumor tissues were assessed using immunofluorescence (Fig. 8A). No significant difference was observed between the NS group and the RLX group (*p* > 0.05). Following TACE treatment, there was a notable increase in PD-L1 expression in the remaining tumors (*p* < 0.01). However, when RLX was combined with TACE, there was a decrease in PD-L1 expression in the residual tumor (*p* < 0.05) (Fig. 8B). In addition, the ELISA results showed that compared with NS group and RLX group, the secretion levels of IL-1β and IL-6 in TACE group were increased, while the levels of IL-1β and IL-6 were decreased after TACE combined with RLX (*p* < 0.01) (Fig. 8C, D). On the contrary, the level of TNF-α secretion in TACE + RLX group was significantly higher than that in TACE group (*p* < 0.01) (Fig. 8E).

### Safety evaluation

HE staining of the lung, spleen, liver, and kidney was demonstrated in Fig. S5A. Seven days after treatment, there were no histological changes in the major organ tissues in the four groups. Prior to treatment and at 1 day, 3 days, and 7 days post treatment, evaluations were performed to assess the hepatic and renal functionalities among the four groups. The findings suggested that the treatment had little influence on hepatic and renal function, and the indices concerning liver and kidney condition retured to their baseline levels after seven days (Fig. S5B).

## Discussion

The ECM of fibrosis after TACE treatment impacts the extent to which DOX penetrates the tumor, restricting DOX effectiveness in tumor cell destruction through chemotherapy and consequently influencing the overall anti-tumor response (Wang et al. 2024). RLX is an effective antifibrogen hormone in humans that inhibits fibroblast activation. In a study by Tabassum Naqvi et al. (2005) using a rabbit model, it was shown that RLX induces upregulation of MMPs, leading to inhibition of fibrin collagen formation. However, due to its short half-life of RLX (~ 10 min), systemically administered RLX usually showed compromised curative effect (Zhou et al. 2021b). To address this issue, our study combines RLX with TACE, which delivers RLX to tumors through hepatic artery targeting rather than systemic administration. Embolization of the tumor blood supply artery at the same time prevents RLX from entering the systemic circulation, which is likely to play a role in longer RLX in tumors.

In our study, we investigated the effects of combining TACE with RLX on reducing fibrosis in the ECM and enhancing the infiltration of DOX into the tumor. Previous studies have demonstrated the significant role of macrophages in the formation of tumor ECM fibrosis (Witherel et al. 2021; Matsuda and Seki 2020). Our results revealed an increase in M2 macrophages and a decrease in M1 macrophages after TACE treatment, potentially facilitating the progression of extracellular matrix fibrosis. The combination therapy with RLX resulted in a reversal of the alterations caused by TACE, leading to an increase in M1 macrophages and a decrease in M2 macrophages. This suggested that RLX might inhibit fibrosis of the extracellular matrix by influencing the polarization of macrophages. Relevant studies have demonstrated that DOX can activate tumor-associated antigens, enhance the infiltration of effector T cells within tumor tissue, trigger an anti-tumor immune response, and enhance the tumor immune microenvironment (Yu et al. 2020; Xie et al. 2024). The findings of this research indicate that the combination of TACE and RLX can elevate the infiltration of CD8 + T cells in the tumor microenvironment, decrease the presence of Tregs, and lower the expression of PD-L1 in tumor cells. These changes may be related to the stronger anti-tumor immune response stimulated by improved penetration of DOX into tumor tissues. Moreover, various cytokines such as IL-1β and IL-6, known to promote tumor development and progression (Li et al. 2022; Kaplanov et al. 2019), are found in the tumor ECM, along with the immune activator TNF-α, which is linked to anti-tumor immune response (Zhu et al. 2021). Our study results indicated that TACE combined with RLX can decrease the secretion of IL-1β and IL-6 while increasing the levels of TNF-α. These results further support the idea that TACE combined with RLX can improve the tumor immune microenvironment. The elevated DOX concentration and improvement immune microenvironment can strengthen the local therapeutic response to tumors and improve the survival outcomes of rabbits with tumors.

Metastasis is the leading cause of death in cancer-related cases (Quail and Joyce 2013). Previous research has suggested that RLX enhances the therapeutic outcomes when combined with other treatments (Binder et al. 2002; Zhang et al. 2023; Hu et al. 2019). However, concerns have been raised regarding the potential of RLX to promote metastasis. In contrast, the findings from our present study indicate that the combination of RLX and TACE treatment actually reduces the occurrence of metastasis in hepatocellular carcinoma.

MMPs is a commonly expressed protein during cancer metastasis, including breast, pancreatic, and hypopharyngeal (Wu et al. 2021; Song et al. 2021). In vitro experimental results showed that although RLX could increase the expression of MMP-9, it did not increase the invasion and metastasis ability of the tumor, suggesting that RLX influenced the tumor microenvironment rather than directly affecting tumor cells. In vivo, RLX can up-regulate the expression of MMP-2 and MMP-9, but no significant change is observed in E-cadherin, which is a crucial marker in the epithelial-mesenchymal transition process during metastasis (Serrano-Gomez et al. 2016). The results from this study appear to be inconsistent with previous research, suggesting the discrepancy may be attributed to variations in the physiological processes of cancer cells. We hypothesized that cancer cell metastasis is a natural progression process, which requires cancer cells to be wholly prepared before initiating metastasis. However, RLX administration is an external intervention. Although the expression of the MMP-9 and MMP-2 protein was elevated, it is likely that most cancer cells have not evolved to metastasis. As a result, this study did not observe enhanced metastasis. The unchanged E-cadherin protein level indirectly supports this hypothesis to some extent. However, further clarification is needed to understand this complex process.

In addition to the results showing that the increase in MMPs did not suggest invasion promotion, which is inconsistent with previous studies, we also observed an increase in the E-cadherin in combination therapy. To better understand of the underlying mechanism, the expression of HIF-1α protein was measured in the tumor tissue. The outcomes displayed a significant decrease in the TACE + RLX group compared to the other three groups, demonstrating that RLX helped alleviate the hypoxic microenvironment in the residual tumor tissue. It is well-established that the activation of HIF-1α and its associated signaling cascade has been linked to an increase in the migration and invasion of cancer cells (Ferrer et al. 2014). Consequently, suppressing the expression of HIF-1α protein through RLX administration effectively inhibited the metastasis of the remaining liver cancer. This finding further supports the synergistic anti-tumor effect of combining RLX with TACE. Furthermore, RLX combined with TACE did not show significant hematological adverse effects or histological toxicity, indicating that RLX did not exacerbate the side effects of the treatment in rabbits.

In conclusion, the combination of RLX and TACE not only improved the antitumor effect of hepatocellular carcinoma but also inhibited metastasis while maintaining biological safety. This innovative concept merits further studies on the clinical applications and on the efficacy on other types of solid malignant tumors.

## Supplementary Information

Below is the link to the electronic supplementary material.

Supplementary file1 (DOCX 11325 KB)

## Data Availability

The data used and/or analyzed during the current study are available from the corresponding author on reasonable request.
