# Supplementary material for: Relaxin combined with transarterial chemoembolization achieved synergistic effects and inhibited liver cancer metastasis in a rabbit VX2 model
Source: J Cancer Res Clin Oncol. 2024 Jul 2;150(7):333. doi: 10.1007/s00432-024-05864-6 (PMC11219380; doi:10.1007/s00432-024-05864-6)
Supplement: Supplementary file 1 — Supplementary file1 (DOCX 11325 KB) [file 432_2024_5864_MOESM1_ESM.docx]

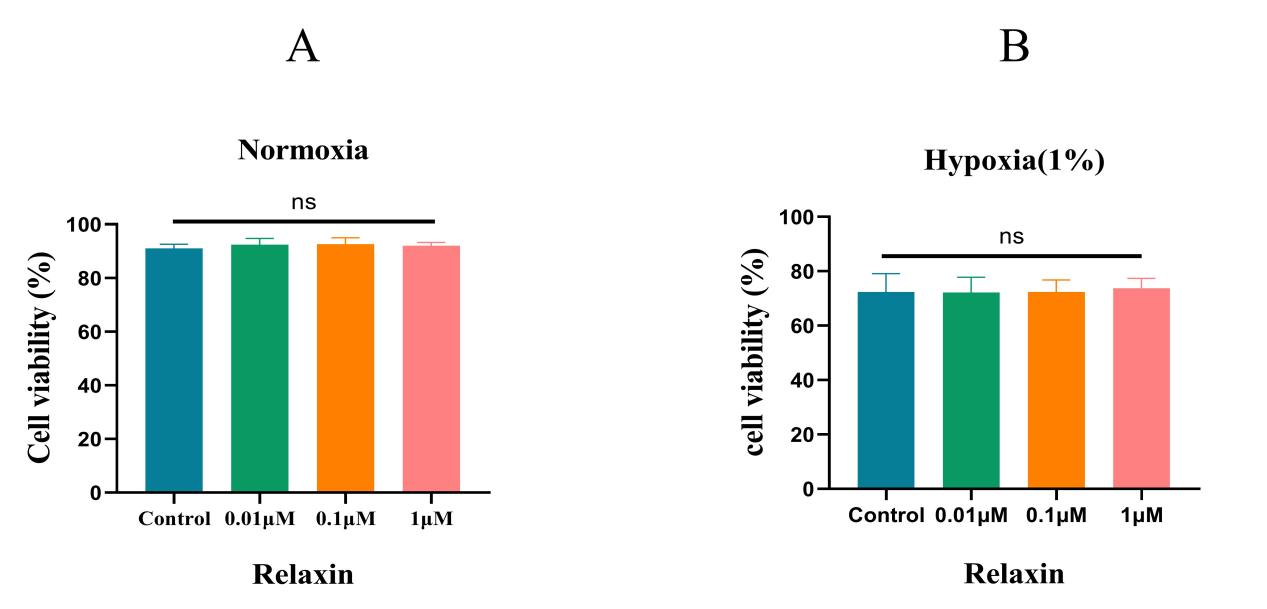


**Fig. S1** RLX did not affect the proliferation of Huh-7 cells.(A), (B) the cytotoxicity of RLX (0, 0.01, 0.1, and 1 μM) on normoxia (A)/hypoxia (B) via CCK-8 on Huh-7 cells (means ± SDs; ns > 0.05)





**Fig. S2** RLX upregulated the expression of MMP-9 but have no impact on the expression of E-cadherin and HIF-1α in Huh-7 cells. (A) Effects of normoxia/hypoxia and administration RLX (0, 0.01, 0.1, and 1 μM) on MMP-9 expression levels in Huh-7 cells; (B) Quantitative analysis of MMP-9 levels in Huh-7 cells; (C) Effects of normoxia/hypoxia and administration RLX (0, 0.01, 0.1, and 1 μM) on E-cadherin expression levels in Huh-7 cells; (D) Quantitative analysis of E-cadherin levels in Huh-7 cells; (E) Immunofluorescence staining of HIF-1α in each group after RLX administration (0, 0.01, 0.1, and 1 μM) (means ± SDs, ns > 0.05, **p* < 0.05, ***p* < 0.01, ****p* < 0.001).

MMP-9, matrix metalloproteinases-9; HIF-1α, hypoxia induicible factor-1alpha; RLX, relaxin.


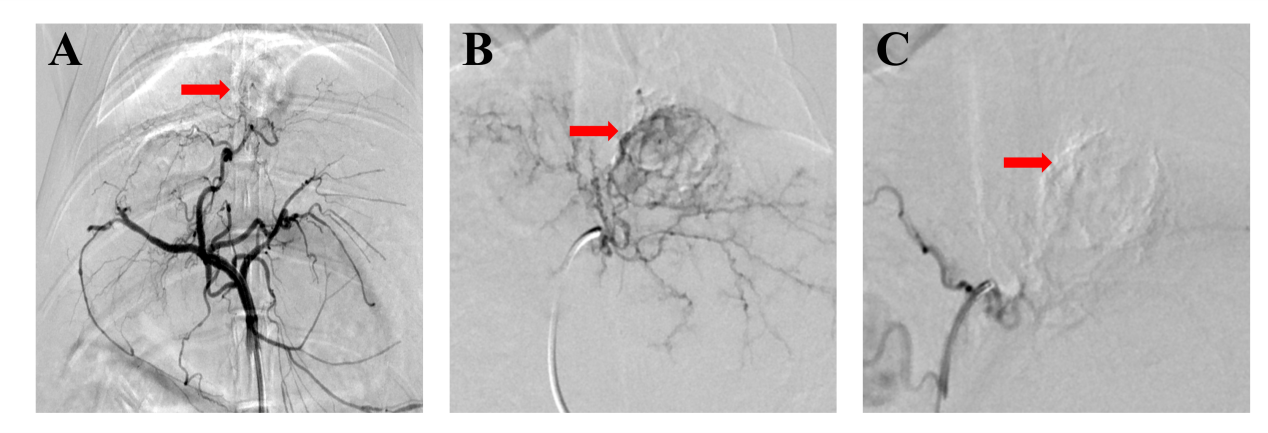


**Fig. S3** Schematic diagram of TACE procedure under the guidance of digital subtraction angiography. (A) Angiography was performed to show the common hepatic artery when the catheter reached the abdominal aorta; (B) The microcatheter was super-selectively inserted into the tumor supplying artery and the angiograph displays the hypervascular tumor (red arrow) in the liver; (C) Angiography after TACE shows the lipiodol deposition (red arrow) within the tumor and the complete occlusion of the tumor-feeding artery

TACE, transarterial chemoembolization


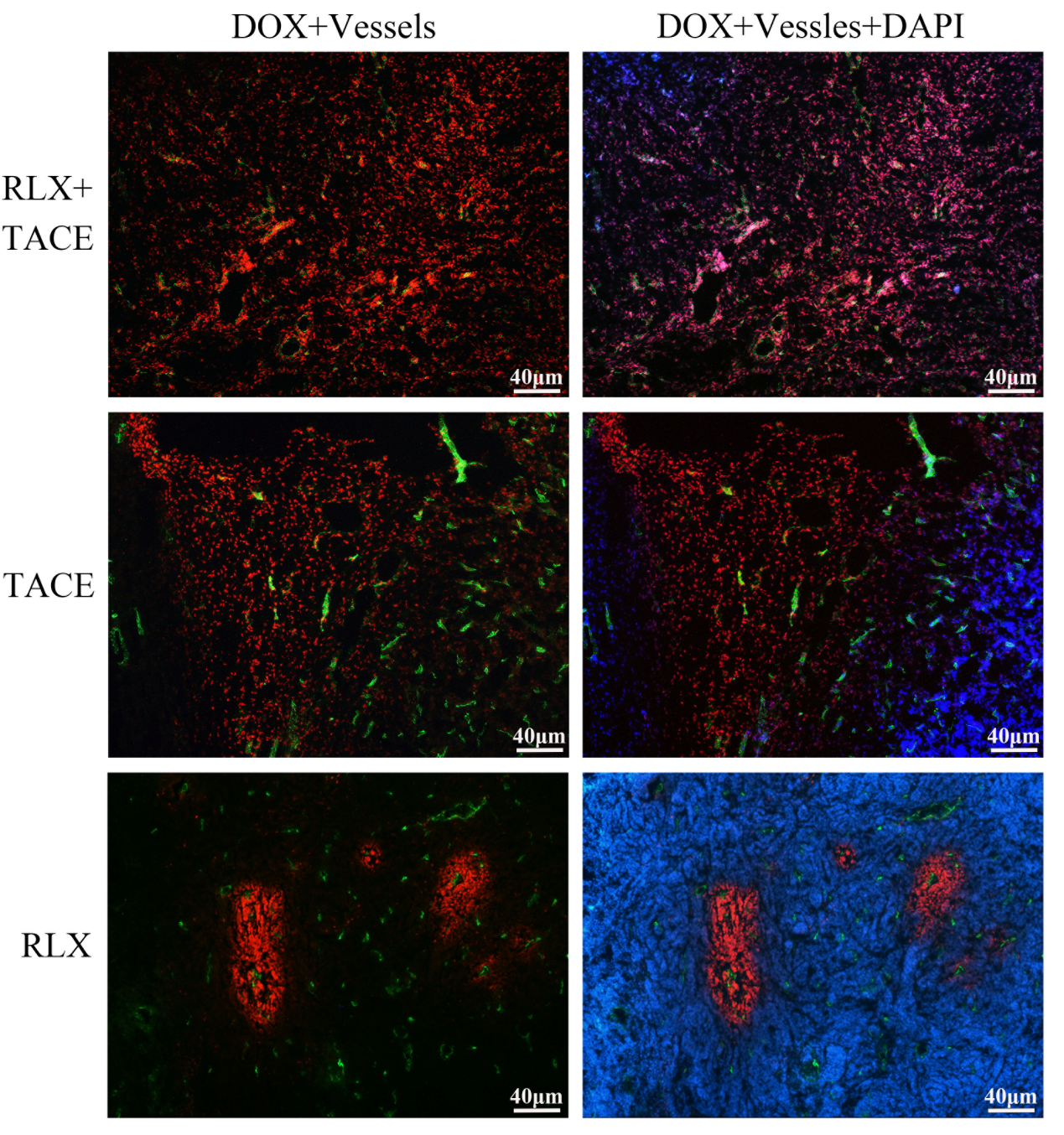


**Fig. S4** Immunofluorescence was performed for image analysis to evaluate the pervasion distance of DOX within the tumor stroma in RLX+TACE group and TACE group. DOX had the natural red fluorescence, the blood vessels were stained with green fluorescence, and the DAPI had blue fluorescence.

DOX, doxorubicin; RLX, relaxin; TACE, transarterial chemoembolization;


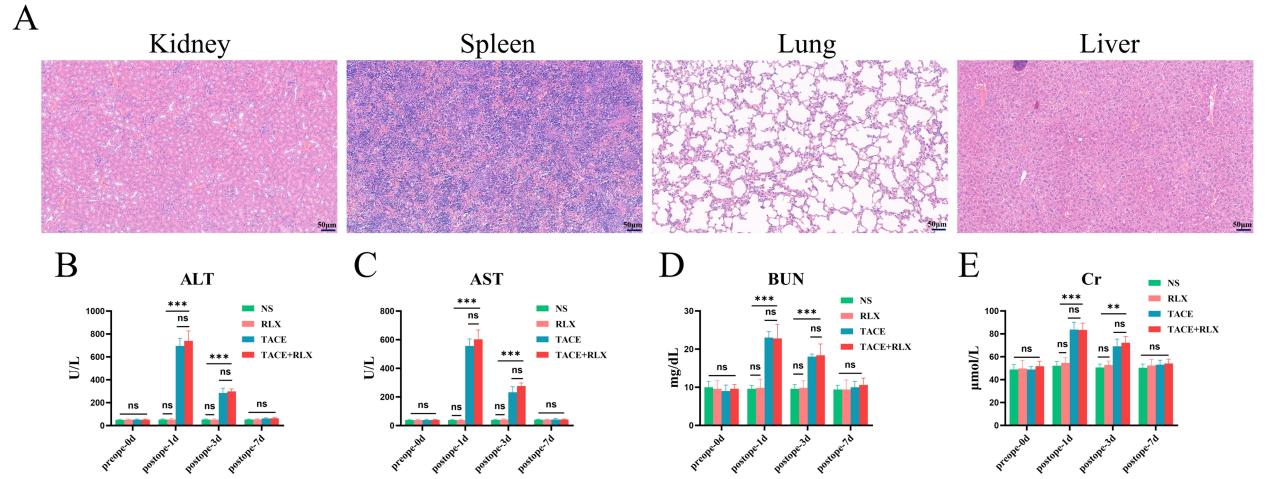


**Fig. S5** Liver and kidney function analysis and H&E staining of the kidney, spleen, lung, and liver. (A) Liver and kidney function analysis in the three groups before and after treatments over time; (B) H&E staining of kidney, spleen, lung, and liver in different groups 7 days after treatments. (means ± SDs; ns > 0.05, **p* < 0.05, ***p* < 0.01, ****p* < 0.001)

RLX, relaxin; TACE, transarterial chemoembolization; NS, normal saline.
